# Creative Research Science Experiences for High School Students

**DOI:** 10.1371/journal.pbio.1000447

**Published:** 2010-09-21

**Authors:** Constance Hammond, David Karlin, Jean Thimonier

**Affiliations:** Tous Chercheurs, Equipe de Recherche Technologique en éducation (ERTé) Hippocampe n°47, INMED and Aix-Marseille II University, Marseille, France; University of California Berkeley/JGI, United States of America

## Abstract

A French research institute raises the bar for public outreach with an educational laboratory that engages 1,000 high school students per year in mini research projects.

The influence of scientific discoveries on daily life has never been greater, yet the percentage of students pursuing careers in science and technology has dropped dramatically in the Western World [1],[2]. Student disenchantment begins even before high school, where students must typically memorize scientific facts and occasionally perform experiments following a strict protocol that teaches abstract concepts with little relevance to daily life [3]–[5]. French high school students, as in other countries, opt out of scientific tracks in the 6th and 7th grades, often selecting scientific courses simply to increase their chances of being accepted at prestigious universities [6]. This passive teaching style squanders children's intrinsic curiosity, imagination, creativity, and fascination with the natural world and forces universities to invest enormous sums in an effort to recover from these lost opportunities [7]–[9]. Offering high school students the means to explore the world the way working scientists do can rekindle their inquisitive nature.

## Tous Chercheurs: A Bioscience Research Program for High School Students

To build bridges between high school students and scientists, our teaching laboratory is located within a research institute of the French medical research council (Inserm), on a scientific campus of the University of Aix-Marseille, France. The institute hosts approximately 1,000 high school students per year for three-day periods to participate in “miniature” research projects. The lab is managed by the non-profit organization Tous Chercheurs—loosely translated as “Researchers, All”—reflecting its philosophy that everyone can be a researcher for at least a little while. Following the success of this program, now five years old, similar initiatives are being planned in other regions of France.

**Figure 1:**
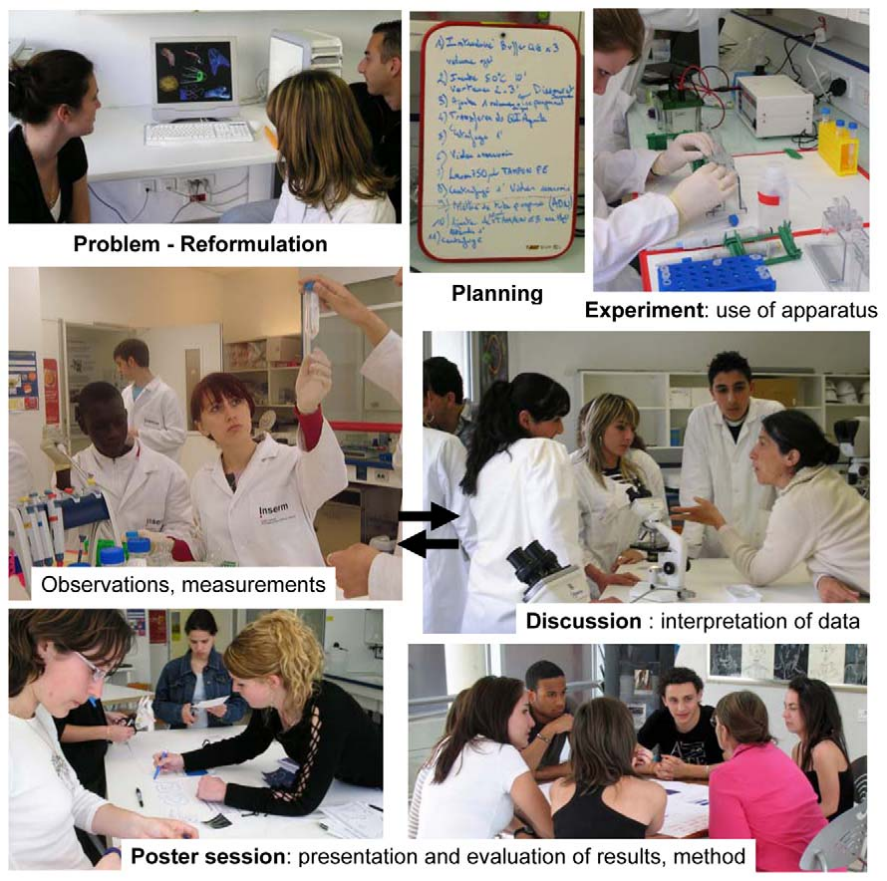
The three-day open-ended investigation including iterative approach between experimentation and interpretation of data (arrows).

The program engages students in open-ended investigations to teach critical thinking and communication skills [10]–[12]. Our approach has two main components: students spend several hours developing a research question (in the context of a well-defined topic), and then a portion of their time post-experiment to consider the problems encountered during their experimentation. They can redo their experiments if necessary (Figure 1). This approach, which is adaptable to any scientific field, relies on six principles (Box 1). This strategy helps pupils learn different aspects of research, including complex and critical thinking [13], the experimental method, and teamwork. In addition, PhD students learn how to teach research in an intuitive and inspiring way.

**Box 1: The Teaching Strategy**

- No pre-selection of students (the entire classroom participates, as well as the science teacher);
- Pupils work in teams, with each team tutored by a PhD student;
- Pupils design, perform, and interpret experiments in a process that is as similar as possible to experiments in a typical research laboratory;
- Pupils perform hands-on experiments (not restricted to computer-based virtual experiments);
- Trials are encouraged and mistakes are not penalized;
- Pupils do not receive grades or exams regarding the research experience. They present their results and discuss their errors much as researchers do among themselves.

## Chronology of a Three-Day Mini Research Project in the Tous Chercheurs Lab

We have created thematic workshops (most lasting three days) within several disciplines. All correspond to the French national curriculum to allow students to focus on the research process rather than on absorbing complex concepts. Our workshops have covered a broad range of research topics and fields, including the uses of fluorescent proteins (molecular biology), response to infection (immunology), brain development and plasticity (neuroscience), and the study and mitigation of aquatic pollution (sustainable development).

Each workshop is separated into three parts: (i) observation, creation, and understanding of a problem, what to study, and how to proceed; (ii) experimentation, quantification, and discussion of the results; (iii) interpretation and critical oral presentation of the results (Figure 1). As in real research, high school students do not know the results of the experiments in advance.

High school teachers organize the class into three to five groups of students, each tutored by a PhD student. Each group independently observes the same biological problem, focusing on two to four slides. For example, in the sustainable development workshop, students are shown two slides describing phenotype modifications of different species of fish living in two types of environments influenced by human activity.

For the discussion during these sessions, tutors neither ask for questions nor provide information unless students ask for it. Students are initially surprised by this approach, but soon become more interactive, sharing their thoughts freely, organizing their thoughts and questions, generating hypotheses, and proposing and designing general protocols to test them, guided by their tutor. During the sustainable development workshop, for example, students identify multiple important aspects of aquatic pollution, including biological, chemical, economical, and sociological perspectives. They must then consider how to identify the impact of pollution on the biological and ionic composition of water and how to minimize it.

When the discussion progresses to a more advanced stage and clear-cut suggestions for avenues of investigation have been made, the tutor explains that resources limit the ability to investigate all of the questions raised and proposes that each of the four groups tests a different, complementary research question, so that all the experiments provide a more complete story that addresses the issue.

Students conduct the experiments they have discussed and designed between the first afternoon and the third morning. The tutor fills in the precise details of the general protocol and teaches them how to read and follow a written protocol, explains why they have to design control experiments, how to use the equipment, suggests that they quantify results, discusses the results with the students, and makes sure that they have dealt with artifacts and interpreted data in order to draw reliable, well-supported conclusions. Though the protocols have been prepared in advance, students may suggest and perform additional experiments to test their ideas. In addition, if a technique fails to yield results (a common situation in the course of research), students interrupt the research project and investigate the likely source of the technical failure with the help of their tutor.

For instance, in the sustainable development workshop, students identify whether the effluent of a waste water plant modifies the bacterial and chemical composition of rivers from water samples taken upstream, at the source, or downstream from the effluent. With microbiological experiments on the three water samples, students identify the phenotypes and metabolism of bacterial colonies grown in Petri dishes, using macroscopic and microscopic observations, respirometry, and polymerase chain reaction (PCR) techniques. Chemical experiments on the same water samples allow comparison of the concentrations of various ions in the three water samples, using colorimetry, photometry, and pH measurements. In addition, students investigate the willingness of an interviewed population to pay for ecosystem preservation (this includes creating an economic survey, interviewing a population on campus, and analyzing the result). Finally, students create slogans for an awareness campaign on pollution based on a tool (metaplan) derived from psychological studies and management tools [14].

Teams prepare slide presentations summarizing their questions, hypotheses, and experimental work and then present their work, explaining the problem investigated, the results obtained, and the conclusions drawn. The director encourages questions and facilitates debate to ensure that pupils understand the work performed by the others. If needed, he explains the question their experiment answers and does not answer, the role of control experiments, and the conclusions they can draw. Then students are sorted into four new “chimera” groups containing at least one student from each of the previous groups. Each chimera group designs a poster that summarizes the multiple investigations performed by the different teams and provides a complete overview of the issue. Assembling the results together reinforces multidisciplinarity and group cohesion and facilitates the subsequent oral presentation.

External researchers (one per chimeric group) are then brought in to listen to the students' explanations of the poster and ask for hypothesis-driven approaches in their explanations (rather than simple recapitulation of the results). They help students critique the poster's title, presentation, figures, and application of the scientific method. Finally, pupils and researchers retrospectively analyze how they could have improved their experimental approach.

## Recruitment of Students and Tutors

This program requires the concerted efforts of researchers (the organizers and assistants) and their labs, PhD students, science teachers, and high school students. Workshops are designed by us (the Tous Chercheurs team) or by groups of PhD students from different scientific fields (such as biology, physics, chemistry, economics) under our supervision. This is considered part of their teaching obligation and their work is generally promoted by a publication [14],[15].

High school teachers have learned about our workshops through word of mouth, and through electronic messages to high schools. Teachers select the research subject six months in advance and organize their lesson plans accordingly. Scheduling the experimental workshop before the theoretical coursework allows students to explore the subject with a more candid, unbiased approach. To ensure teachers that the PhD students can take their place in the lab, the lab's team explains how the plan will work and how pupils will be taught to conduct experiments. Participating teachers are highly motivated to manage the time required for the workshops by collaborating with other teachers at their school. For example, teachers may swap duties for the days needed in exchange for an invitation to attend the course.

Tutors are recruited by advertisement through PhD student associations, trained before their first workshop, and are paid for the sessions. They gain valuable teaching experience for their CVs, and a better understanding of the research process. It is so completely different from their previous experience as lecturers in a passive instructional role, that it often takes some time before they can fully engage the students in active learning. The high school students are actually helpful in that they may look up to the tutors (often closer in age than their own instructors), and come to mimic many of the researchers' behaviors. Researchers who come to the lab at the end of the workshop for the students' presentations are easily recruited from campus labs thanks to their interest in interacting with students and because they enjoy explaining their day-to-day life as researchers.

We have not encountered any problems with keeping students interested. They enjoy the chance to actively participate during the school day, to work as a team, and to test their ideas, experiment freely, and engage in discussion with their tutor. The layout of the lab is also very important. The benches of our lab are not aligned in rows, which hinder efficient teamwork, but are easily moved, allowing many people to engage in face-to-face discussions. Students can move freely within the lab and to their offices opposite the lab, where they have access to computers and whiteboards. They also have access to an outdoor terrace to relax under the Mediterranean sun.

Though evaluation of the program will be complete by the end of 2012 [16], we have results from one cohort of high school students who participated in the program for three years (2006–2008). Seventy-four percent of those who passed the French baccalaureat (equivalent of A level in the UK or college entrance exam in the USA) are majoring in science at the science or engineering universities, and 5% are enrolled at liberal arts universities.

A few other centres offer high school students the opportunity to conduct experimental science in a dedicated laboratory on a university campus. Israel pioneered this concept with The Belmonte Science Center for Youth (http://www.belmonte.huji.ac.il/), and at least six others have since been created in Europe. These include XLAB (http://www.xlab-goettingen.de/), Life Lab (http://www.lifelab.de/), and Gläsernes Labor (http://www.glaesernes-labor.de/)(Germany), Open Lab (http://www.viennaopenlab.at/) (Austria), House of Science (http://www.houseofscience.se/) (Sweden), and Petnica Science Center (http://www.psc.ac.yu/eng/) (Serbia). They all provide excellent equipment and mentoring by scientists. Although they differ in the duration of the workshop (four hours to several days), whether or not they pre-select students and in their educational approach, all share the common goal of encouraging high school students to choose scientific careers.
